# The Role of Nanoparticles in Accelerating Tissue Recovery and Inflammation Control in Physiotherapy Practices

**DOI:** 10.7759/cureus.73540

**Published:** 2024-11-12

**Authors:** Jyothsna Volisha Cardoza, Zeeshan Ali, Simi Simon, Darshni Thakkar, Sudhan S George, Samuel Paul Isaac

**Affiliations:** 1 Pharmacology, Krupanidhi College of Physiotherapy, Bengaluru, IND; 2 Physiology, Krupanidhi College of Physiotherapy, Bengaluru, IND; 3 Biochemistry, Krupanidhi College of Physiotherapy, Bengaluru, IND; 4 Physiotherapy, Krupanidhi college of physiotherapy, Bengaluru, IND; 5 Physiotherapy, Krupanidhi College of Physiotherapy, Bengaluru, IND

**Keywords:** inflammation, nanoparticles, pain management, physiotherapy, tissue recovery

## Abstract

Physiotherapy has significantly evolved since its inception in the late 19th century, expanding into various specializations such as sports, neurology, and wound care. Its primary goal is to restore or enhance bodily functions through therapeutic interventions, aiding in conditions ranging from injuries to chronic pain. Tissue recovery, which involves repair and regeneration, is a critical aspect of physiotherapy. This natural process is influenced by factors like inflammation and injury severity. Nanotechnology, a relatively recent advancement, has transformed medicine, including wound care, through innovations in drug delivery, diagnostics, and anti-inflammatory treatments. Nanoparticles, owing to their small size and enhanced bioavailability, play a crucial role in improving drug delivery, increasing the efficacy of treatments, and promoting faster recovery. In the context of tissue healing, nanoparticles aid in cell proliferation, inflammation control, and scar reduction, among other therapeutic benefits. They are increasingly used in physiotherapy applications, to support tissue regeneration and inflammation management. This review examines the role of nanoparticles in physiotherapy, with a focus on their application in wound healing, muscle recovery, and inflammation control. It discusses various in-vitro and in-vivo studies that have explored the therapeutic potential of nanoparticles in these domains, providing insights into their mechanisms of action and effectiveness in promoting tissue regeneration and managing inflammation in physiotherapy settings.

## Introduction and background

Background on physiotherapy and tissue recovery

Tissue recovery means repairing any tissues that may have suffered damage after injury. Physiotherapy is the use of physical procedures, such as exercise or manual therapy, backed up by scientific evidence, to restore motion, relieve pain, strengthen the affected area, and allow for maximum recovery and minimal risk of complications during the rehabilitation stage [1]. Sports, wound care, neurology, geriatrics, cardiopulmonary, pediatrics, and orthopedics are just a few of the specializations included in this field. Neurological rehabilitation is a field that is developing quickly. Physiotherapists perform their duties in a variety of venues, such as skilled nursing homes, outpatient clinics, health and wellness centers, hospitals for rehabilitation, private residences, colleges, educational institutions, hospitals, industrial workplaces, fitness centers, and sports training facilities. The goal of physiotherapy, which has its roots in movement sciences, is to improve or restore the functionality of several body systems. The field incorporates a wide range of both physical and physiological therapeutic interventions and assistance to improve well-being, existence, and the excellence of eternal life [1,2]. Its main goals are to maximize movement potential and quality of life using intervention, regeneration, therapy, prevention, and rehabilitation. Interferential therapy, ultrasound, short-wave diathermy, wax therapy, and muscular strengthening exercises are some of the pain-relieving techniques used in physiotherapy. It helps people who are impacted by disease, injury, or disability regain their range of motion and functionality. From electrical stimulation, heat, and water-based applications to heat electrical stimulation, and water-based applications, physiotherapy has changed dramatically over time. It is the most widely recommended course of treatment for a number of ailments and injuries, such as persistent pain, sports and auto injuries, and mobility issues [2].

Restoring tissue functionality and architecture following a wound is a natural, ongoing process in living things called tissue regeneration. Repair and regeneration are its two main components. In restoration, collagen is deposited to produce a scar; in regeneration, lost or damaged tissue is replaced with intact cells and tissue. The procedure varies according to the degree of injury and the tissue's capacity for regeneration. Scars and collagen deposition are necessary for serious injuries, which permanently change the structure of the tissue. For superficial wounds, recovery entails surface epithelium regrowth. As severe fibrosis can result from persistent inflammation, tissue healing is crucial to preserving general health [3]. In general, inflammation helps the host respond to foreign objects, infectious agents, or tissue damage and is a necessary step before tissue structure and function are restored. Both the periodontium hard and soft tissues suffer significant damage as a result of the persistence and accumulation of inflammatory cells in the periodontal tissues. It is conceivable for the inflammation acute phase to self-limit and resolve, even though the chronic phase of inflammation ultimately results from the ongoing activation of the acute response. Understanding the cellular and molecular mechanisms behind the resolution of inflammation has come a long way in recent years [4].

Nanotechnology in medicine

Applications of nanotechnology, a relatively young scientific field, can be found in many areas of biology and medicine, including food technology, bioengineering, biochemistry, biophysics, and medical and biomedical sciences. In the last forty years, advances in nanobiotechnology have been demonstrated in the diagnosis and treatment of many diseases, drug discovery, personalized medicine, cancer treatment, pharmaceutical discoveries, and the development of innovative medical instruments and techniques. Numerous medical application streams are combined by nanomedicine, such as pain management, disease treatment, diagnosis, prevention, human health improvement, and nanoscale technology against traumatic damage. And disease treatment alternatives [5-7]. By providing targeted and regulated administration to particular bodily regions, such as tumors, inflammatory tissue, and contaminated areas, nanotechnology has completely changed the way drugs are delivered to patients. This decreases negative effects and lowers the need for medication. Drugs as medical therapy have proved to have more stable characteristics during drug delivery and high solubility in the presence of nanoparticles (NPs). This increases their efficacy in treating diseases. Additionally, by improving distribution and absorption, they increase bioavailability, enabling lower dosages and less toxicity. Medical nanorobots for antitumoral responses and wireless surgeries against numerous diseases are being developed, and drug delivery systems are being modified using the rules of nanoscale. Nanotechnology may be used to diagnose and treat blood-related conditions as a result of the development and testing of mechanical red blood cell technologies like respirocytes [8-10]. 

Nanotechnology promises huge strides in physiotherapy through the improvement of therapeutics, tissue engineering, and imaging techniques. Nanomaterials use the principle of targeted drug delivery systems which ensures the drugs administered only go to areas of inflammation following injury hence reducing systemic toxicity and maximizing efficacy. The sustained release of pain medications enhances pain control thus extending the pain-free period for patients of physiotherapy [7,8]. Also, NPs help in enhancing cellular proliferation and the repair of the extracellular matrix, hence promoting faster recovery from injuries to the skeletal system. Apart from MRI, where contrast agents that contain such nanomaterials are used and this gives results that are more accurate than before, other applications of these images include prognosis of diseases and follow-up of the physiotherapy interventions. All these advancements bring the most recent developments of nanomedicine into physical therapy regimens, rendering effective and quick healing processes specific to the patient’s needs, and shortening the healing times [8,9].

The objective of this review is to provide an in-depth discussion on the role of NPs in tissue restoration or wound healing as well as inflammation management with the help of physiotherapy and its techniques. Different in-vitro and in-vivo studies related to tissue regeneration and inflammation control were discussed elaborately.

Search strategy for literature review

Literature searches were conducted in different digital databases (Google Scholar, Scopus, PubMed, Web of Science, and Embase). The following search terms were used for the literature search: (“nanoparticles,” “tissue recovery,” “inflammation control,” “physiotherapy,” “nanomedicine,” “muscle repair,” “anti-inflammatory nanoparticles,” “wound healing,” “targeted drug delivery,” “nanoparticle clinical trials”). The articles were primarily screened to assess appropriate for inclusion according to the metrics with studies published in peer-reviewed journals, clinical trials, in-vitro and in-vivo studies involving NPs in tissue recovery and inflammation management, studies from the last 10 years were downloaded to identify the significance of the investigation for scientists. During selection, the following data were eradicated including types of NPs, their therapeutic applications, outcomes in tissue recovery and inflammation control, clinical trial results, and the methods used in physiotherapy settings. All the suitable and downloaded articles in the study were analyzed, examined, and articulated meticulously. Articles without experimental validation, review papers not focusing on physiotherapy or tissue recovery, and studies published before 2010 (unless seminal), were excluded from the study performed.

**Figure 1:**
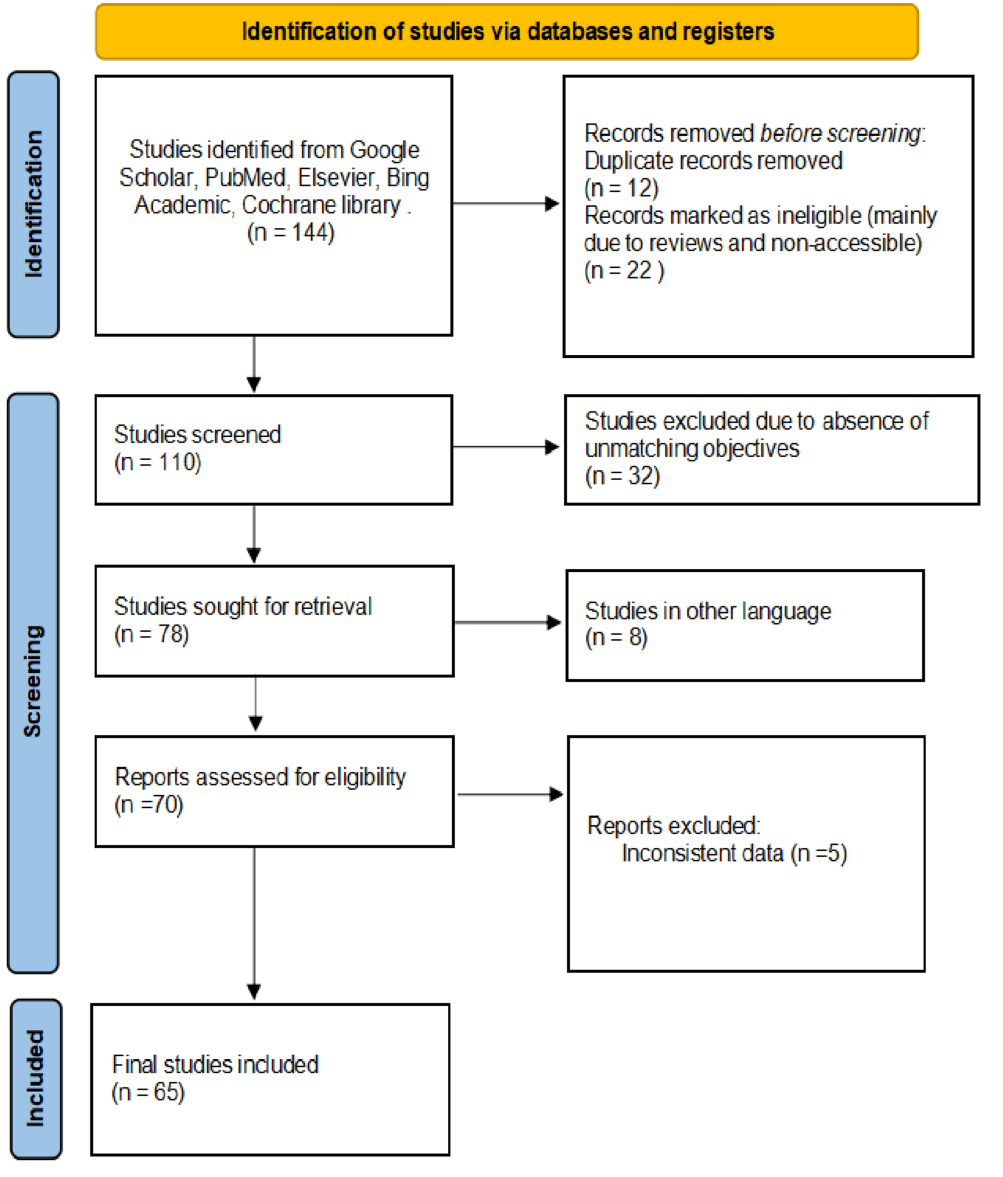
PRISMA flowchart

## Review

NPs: properties and therapeutic potential

Overview of NPs

NPs are microscopic particles that differ in size from 1 to 10 nm in any manner and have characteristics similar to those of molecules and bulk materials. Because of their substantial surface-to-volume ratio, they are referred to as the “bridge” between macroscopic and microscopic structures [11]. They have a broad range of components and are extremely flexible, which results in sluggish sedimentation rates. Depending on their shape, they can have a 1D, 2D, or 3D structure. They are classified as lipid-based, carbon-based, metal-based, polymeric-based, ceramic-based, and semiconductor-based NPs. The two primary techniques to formulate NPs are top-down and bottom-up. Chemical vapor deposition, sol-gel, physical vapor deposition, radio frequency plasma, thermolysis, pulsed laser, and solution combustion processes can all be used for studying NPs [12-15]. X-ray photoelectron spectroscopy (XPS), transmission electron microscopy (TEM), atomic force microscopy (AFM), scanning electron microscopy (SEM), polarized optical microscopy (POM), infrared (IR), Brunauer-Emmett-Teller (BET), X-ray diffraction (XRD), Raman spectroscopy, and Zeta size analyzer are some of the techniques utilized for analyzing NPs [16,17].

Mechanisms of Action in Tissue Recovery

Through the development of NPs with distinctive physicochemical properties, nanotechnology has greatly revolutionized wound care. The hemostasis, antimicrobial activity, inflammatory regulation, and cell proliferation associated with wound healing have all been demonstrated by these materials' efficacy. Technology that combines gelatin and sodium alginate with NPs can improve these characteristics. Cell adhesion and proliferation are aided by bionic nanofiber scaffolds that imitate the epidermal cell matrix of the skin. Owing to their innate properties that accelerate the healing procedure, NPs are essential in applications involving wound healing [18]. To provide a continuous and controlled release of therapeutic molecules for improved wound healing results, researchers are using bioengineered drug delivery systems. The intrinsic advantages of polymer nanomaterials, metal and metal oxide NPs, and other nanotherapeutics have led to their widespread use in the treatment of chronic wounds. Prior to in-vivo application, though, it is critical to take into account the possible toxicity of metal-based nanotherapeutics [19]. Antibiotics and growth hormones added to polymeric nanostructures have the potential to reduce wound infection and speed healing, making them useful for skin regeneration and wound amelioration [20,21]. Especially in chronic wounds that remain in the inflammatory phase, nanomaterials are essential to the healing process. Their porous nanosponge-like properties, which help in exudate absorption, skin wound surface protection, and blood clotting acceleration, are examples of their hemostatic properties. Moreover, they have electrostatic adsorption qualities that promote fibroblast adhesion and blood cell recruitment. The effects of inorganic NPs on thrombosis and platelet adhesion have been investigated, including silica and carbon nanotubes. When applied during the hemostatic phase, nanomaterials function as active wound dressings and speed up the healing process more than conventional dressings. Antibacterial and antioxidant qualities are provided by non-bioactive components such as chitosan NPs, metal ions, and silica NPs [22,23].

NPs in Inflammation Control

The synthesis of delicately assembled nanomedicines has been one of the main ways that the comprehension of the inflammatory response has contributed to novel techniques in the treatment of inflammation. Systemic toxicity and off-target organ side effects can be addressed by engineering NPs to target particular organs. By phagocytosing inflammatory sensors or macrophages, these NPs target and control the expression of pro- and anti-inflammatory chemicals. By concentrating on effector cells, especially those that present antigens, they may also enhance cellular response or immunological tolerance. Nanocarriers can target these cells either passively or aggressively due to their modulability. Better ways to combat the adverse reactions of traditional medications have emerged as a result of the discovery of more potent anti-inflammatory nanomedicines [24,25]. One group of steroid hormones called corticosteroids is essential for immunological response, inflammatory control, and stress response, among other physiological functions. Glucocorticoids are powerful anti-inflammatory drugs, such as hydrocortisone, prednisolone, dexamethasone, and budesonide; nevertheless, their side effects restrict their therapeutic application [26,27]. To prevent cyclooxygenase proteins from functioning, non-steroidal anti-inflammatory drugs (NSAIDs) are used to treat inflammation; however, excessive dosages may cause toxicity. Chitosan-poly(γ-glutamic acid) NPs loaded with clofenac, which demonstrated minimal cytotoxicity for human macrophages, are one type of NP that has been designed to combat these negative effects. For the treatment of disorders associated with inflammation, anti-inflammatory mediators such as IL-10 have been created; more effective targeting has been observed using IL-10 coupled to nano-sized phosphatidylserine-containing liposomes (PSL). Researchers are also looking into the possibility of treating inflammation with anti-inflammatory peptides like KAFAK [28-30]. The summary of mechanisms is shown in Table 1.

**Table 1:** Mechanism of actions that nanoparticle adapts in tissue recovery

Mechanism	Nanoparticle technology	Key benefits
Wound Healing	Gelatin and sodium alginate combined with nanoparticles, bionic nanofiber scaffolds	Cell adhesion, proliferation, controlled therapeutic release, antimicrobial activity, haemostasis
Inflammation Control	Engineered nanoparticles targeting organs, corticosteroid-loaded nanoparticles, NSAIDs NPs	Targeted delivery, reduced systemic toxicity, enhanced cellular response, anti-inflammatory effects
Chronic Wound Management	Chitosan nanoparticles, metal ions, silica nanoparticles, nanosponge-like materials	Antibacterial, antioxidant properties, blood clotting acceleration, fibroblast adhesion, exudate absorption

Forms of application of NPs in physiotherapy

Topical Applications

Wound healing and reconstruction of the skin are finding expanding applications for nano-DDSs, such as lipid NPs, inorganic NPs, nanofibrous structures, polymeric NPs, liposomes, and nanohydrogels. Hydrogels are porous, three-dimensional networks that are elastic and formed of hydrophilic polymers, usually with a moisture content of at least 10%. They respond to stimuli like as pressure, pH, temperature, ionic charge, or antigens, among other things, and they revert to their initial state when the stimulation stops. The medical industry uses these “smart” materials for biosensors, tissue engineering, cell treatments, medication delivery, wound care, and tissue engineering. They provide advantages such as preventing complications, accelerating the healing of wounds, improving patient satisfaction, and improving therapeutic outcomes. Hydrogels possess several attributes such as comfort, moisture retention, gas permeability, exudate absorption, biocompatibility, and non-adhesiveness. Antibiotics, growth factors, NPs, and stem cells are examples of active substances that can enhance their intrinsic qualities [31,32]. Silver NPs (Ag-NPs) have been popular in wound dressing polymers, and their antibacterial qualities have made them a preferred choice for NP dressing. In addition to controlling microbial growth, these NPs aid in wound healing. Still, they have encountered difficulties such as resistance to microorganisms. Reviewing Ag-NP-functionalized wound dressings and their potential to transform wound healing, this paper addresses therapeutic techniques. To help with the understanding of Ag-NPs' application in wound care, it also covers the physiological processes of skin and wounds [33]. A widely used method to fabricate homogeneous, extremely porous polymeric nanofibrous scaffolds with characteristics akin to endothelial cells (ECM) is electrospinning. These scaffolds' enormous surface area and small pores make them appealing for wound healing. AgNPs were used to modify the surface of poly(dopamine methacrylamide-co-methyl methacrylate) (MADO), which was electrospun into a fibrous scaffold. High antibacterial activity against *Escherichia coli*, *Staphylococcus aureus*, and *Pseudomonas aeruginosa* was demonstrated in vitro experiments, and collagen nanofibers loaded with AgNPs exhibited quicker wound healing. In vivo experiments indicated quicker wound healing for AgNP-loaded collagen nanofibers. AgNP-loaded covalently crosslinked alginate fibers demonstrated potential for wound healing as well [34,35].

Injectable NPs

An investigation explored the possibility of using pH-sensitive calcium-based NPs to improve cutaneous wound healing. To treat the wound bed, the researchers applied topically or intravenously several populations of NPs that they had manufactured on plates covered with collagen. It was observed that the intravenous administration of the NPs led to a dose-dependent contracture of a fibroblast-populated collagen lattice, an increase in the pace of wound healing, and an increase in fibroblast calcium absorption in vitro. The rate of fibroblast growth was also accelerated by the NPs. As a result of releasing ionized calcium into the wound bed, intravenously injected calcium-based NPs have been shown to reduce the size of open wounds by contracture. The first study to show the potential therapeutic effects of calcium-based NPs has important ramifications for the management of wounds [36].

NPs in Combination With Physical Modalities

Considering nanomaterials promote healing and have antimicrobial qualities, metal NPs including zinc, gold, and silver are utilized to treat diabetic-induced wounds. In both in vitro and in vivo experiments, these combinations have demonstrated encouraging outcomes when paired with biomaterials such as chitosan and bacterial cellulose. Toxicities and synthesis techniques are still problems, though. Although there are still obstacles to overcome, dual therapy employing biomaterials and metal NPs has demonstrated encouraging outcomes in the healing of diabetic wounds [37]. Photodynamic treatment (PDT) was found to considerably reduce wound size and bacterial counts in animals with infected skin wounds, according to a comprehensive assessment of 19 publications on the subject. By encouraging wound closure and eliminating germs, PDT also expedited the healing process. Most of the research used Pseudomonas aeruginosa or methicillin-resistant Staphylococcus aureus to inoculate wounds on mice and rats [38].

Role of NPs in tissue recovery

NPs in Muscle and Tendon Recovery

The prevalence of tendon injuries is rising worldwide, yet because of poor perfusion, sluggish healing, and the creation of scar tissue, existing tendon restoration procedures have not produced much progress. Present tendon TE scaffolds lack time-dependent dimensions and biomechanical forces, and they are static and non-animating. In the future, techniques such as 3D or 4D bioprinting may yield smart tendon or ligament scaffolds capable of self-in-vivo regulating, self-animating, and self-healing, thereby paving the way for smart orthopedics. Successful tendon augmentation methods require more study and clinical trial investigations [39,40]. Blocking ATP Synthesis in bacteria and denatured DNA, silver NPs have been discovered to be antimicrobial agents. Aside from that, their antiflogistic properties hasten the healing of burn wounds. By encouraging cell proliferation and boosting the formation of collagen and proteoglycan, silver NPs expedited tendon repair in a study by Kwan et al. Additionally, they decreased adhesions and the production of scar tissue [41]. Using controlled injections of biocompatible NPs, Empson and the team investigated the in-vitro biomechanical and cellular response of NP therapy for injured connective tissues, with the hypothesis being that this would improve matrix mechanical characteristics. Finding that NPs can regionally reinforce impaired connective tissues, they employed swine skin as a prototype for repairing target tissues [42]. Furthermore, recent investigation has demonstrated that NPs can improve the mechanical characteristics of natural polymer matrices like chitosan, regenerated cellulose, and decellularized pig diaphragm tendon, as well as modify cellular responses [43].

NPs for Cartilage and Joint Repair

Osteoarthritis (OA), a disorder marked by microscopic changes and degradation of joint functionality, has prompted the medical and physiotherapeutic community to use nanotechnology to develop targeted pain medications using NP drug delivery systems. The efficiency of intra-articular drug administration is enhanced by these methods, as self-assembled HA-NPs target CD44 and prolong the half-life of IL-1RA in rat cartilage. Drug recognition in the extracellular matrix (ECM) and joint cavity can also be enhanced by amine-terminated polyamidoamine (PAMAM) dendrimers. Changes in the regional surroundings brought on by the disease or external stimuli may encourage the targeting of NPs. When anti-inflammatory drugs are administered specifically to reduce side effects and the therapeutic dose, they are more successful in treating osteoarthritis [44,45]. Kang et al. developed thermally sensitive polymer nanospheres for simultaneous and independent dual medication delivery, while Deloney et al. designed hollow solid thermoreactive NPs to stop post-traumatic OA progression in rats. Remarkable anti-inflammatory and cartilage-protective properties are exhibited by dopamine NPs. NIR (near-infrared) light has low absorption, less scattering, and less autofluorescence, and it can pass through tissues [46,47]. Tailored and precision therapy has demonstrated the great efficiency of microcellular drug delivery methods, including liposomes, gene therapy, and hydrogen peroxide-sensitive nano micelle (PLGA-SeSe-mPEG) [48]. Osteoarthritis can now be effectively treated using an empty self-assembled hyaluronic acid NP (HA-NP), a novel therapeutic agent. By expressing CD44, HA-NP enhances cellular absorption by blocking receptor-mediated cellular uptake of low-molecular-weight HA. Additionally, it exhibits long-term retention capacity in knee joints as well as resistance to digestion. According to the study, HA-NP specifically targets CD44, a crucial gene linked to degenerative cartilage conditions [49].

NPs in Skin and Wound Healing

NPs serve an excellent role in wound recovery by transporting chemicals produced at the injury site, such as nitric oxide (NO). However, the absence of efficient delivery molecules limits their practical application [50]. Regenerative medicine has new approaches thanks to nanotechnology, but wound healing is still a difficulty. Nanomaterials can improve burn therapy and delay wound healing. Examples of nanomaterials are metal NPs such as gold, silver, and zinc. These special NPs, which are usually 10-9 nm in size, have bacteriostatic and bactericidal properties together with low in-vivo toxicity [51]. Studies have revealed that topical use of diazeniumdiolate (NO-releasing NP) in diabetic mice expedited wound closure, increased blood vessels and fibroblasts, and ordered collagen content. NO-NP therapy enhances growth factors and anti-inflammatory cytokines while hastening wound closure [52]. In-vivo experiments in diabetic rats demonstrated that topical treatment of recombinant human epidermal growth factor (rhEGF) integrated poly(lactic-co-glycolic acid) NPs expedited wound recovery, enhanced epithelization, and boosted fibroblast proliferation [53]. Bioactive phyto compounds with antibacterial and wound-healing attributes, including curcumin, which possesses antibacterial, anti-inflammatory, and antioxidant qualities, have also been delivered by nanotechnology [54,55]. Wu et al.'s invention of nanoscaffold dressings, which impregnate bacterial cellulose nanofiber with silver NPs (AgNP), can help reduce infection and microbial load [56]. A comparative role of NPs in muscle and tendon recovery, joint repair, and wound healing has been shown in Table 2.

**Table 2:** Comparative role of nanoparticles in muscle and tendon recovery, cartilage and joint repair, and skin and wound healing

Aspect	Nanoparticles in muscle and tendon recovery	Nanoparticles for cartilage and joint repair	Nanoparticles in skin and wound healing
Key Challenge	Poor perfusion, slow healing, and scar tissue formation in tendon injuries	Microscopic changes and degradation of joint functionality (e.g., osteoarthritis)	Delayed wound healing and burn therapy
Nanoparticle Function	Silver nanoparticles aid in cell proliferation, collagen formation, and scar tissue reduction	HA-NPs target CD44, enhancing drug retention and reducing inflammation in joint tissues	Nanomaterials (e.g., gold, silver, zinc) enhance bacteriostatic, bactericidal properties
Innovative Techniques	3D/4D bioprinting for smart tendon/ligament scaffolds, self-regulating and healing	Dual medication delivery systems (e.g., thermally sensitive polymer nanospheres)	Nanoscaffold dressings with silver nanoparticles reduce infection and microbial load
Specific Benefits	Regionally reinforces impaired tissues, reduces adhesions, improves matrix characteristics	Enhances drug delivery in cartilage, prolongs drug half-life, targets degenerative conditions	Speeds wound closure, enhances blood vessel formation, promotes fibroblast proliferation
Therapeutic Application	Tendon repair, reducing scar tissue, improving biomechanical characteristics	Osteoarthritis treatment, cartilage protection, post-traumatic joint recovery	Diabetic wound recovery, infection reduction, anti-inflammatory and antioxidant delivery

Role of NPs in inflammation control

NPs As Anti-inflammatory Carrier

Excessive reactive oxygen species (ROS) and inflammation in wounds are associated with delayed wound healing. Anti-inflammatory and antioxidant medications applied topically help accelerate the healing of skin lesions, particularly those caused by diabetes and chronic conditions [57]. It has been explained that the combination of Au NPs with anti-oxidative small molecules (α-lipoic acid and epigallocatechine gallate) enhances angiogenesis and reduces inflammation, hence promoting wound healing. Additionally, loaded medicines' skin absorption can be boosted by Au NPs. Furthermore, to work in concert to expedite wound healing, nanocomposite materials with anti-inflammatory and antioxidative properties have been formulated [58]. To help treat infected wounds, Mao et al. created a multipurpose hydrogel with antibacterial, anti-inflammatory, and antioxidative qualities. Applying the multifunctional nanocomposite hydrogel topically to wounds demonstrated enhanced granulation tissue thickness and faster wound healing [59].

Targeted Delivery of NPs to Inflammatory Sites

A new wound healing therapy, BR~PolyET-NC gel, has been designed using a three-level, three-factor Box Behnken design. The gel, loaded into Carbopol gel, showed improved biopharmaceutical performance and wound-healing potency in adult BALB-c mice. The gel's in-vitro investigation disclosed uniform consistency, well-spreadability, and extrudability, making it appropriate for topical use. The gel also reduced microbe colony count and monocyte and lymphocyte counts, demonstrating its effectiveness in wound closure and healing [60]. In physiotherapy practice, the use of nanotechnology-based gels like those that achieved more than 99% wound closure by day 15 in vivo, might be more beneficial in rehabilitation and recovery processes. Such gels can be used to aid in the repair of the tissues in the skeletal muscle system, in wound care after surgery, or in treating pressure sores [59]. These can help to achieve restoration of function and recovery of the areas of mobility by enhancing the healing process and limiting scar formation. In addition, these gels may also be used alongside therapeutic exercises and other procedures so, that the optimal environment for tissue repair is maintained, and overall rehabilitation time is shortened. This demonstrates the collaboration of cutting-edge biomedicines and physiotherapeutic techniques [58-60].

Role of NPs in Reducing Oxidative Stress

 Curcumin is one of the phenolic phyto components that has antioxidant qualities that shield plants from oxidative damage. Turmeric comprises a phyto polyphenol called curcumin, which has neuroprotective, anti-inflammatory, and anti-cancer activity. It aids in the regeneration and repair of neurons by lowering oxidative stress in neuronal cells. Liposomes coated with curcumin have been produced to reduce oxidative stress, protect ankle joints from inflammation, and cross-link the brain-brain barrier [61]. In physiotherapy practice, the therapeutic agent curcumin is gaining prominence as an adjunct to conventional rehabilitation, especially in the management of injuries and chronic ulcers. It acts effectively on the affected areas because of its high anti-inflammatory, antioxidants, and antimicrobial effects which promote healing and lower the oxidative stress present in injured tissues. It is used in the form of gels, creams, or even nanotechnology-based formulations, which can promote healing, decrease inflammation, and prevent hypertrophy when applied to the skin. In the case of musculoskeletal injuries, while measuring rehabilitation, curcumin helps in controlling pain and inflammation. This improves the physiotherapeutic outcomes in recovering patients presented with chronic wounds, inflamed joints, or wounds [62].

## Conclusions

With enormous potential to improve results, the application of NPs, nanofibers, and nanosheets in wound healing is a quickly developing topic. These substances can distribute bioactive compounds, encourage tissue repair, and fend off infections. They may be utilized in the formation of innovative wound dressings and biomedical devices for targeted therapy, while assurance and potency are being investigated. To sum up, the application of NPs in physiotherapy can radically change how one treats patients. Target-specificity, anti-inflammatory action, and enhancement of tissue repair are just some of the reasons they are used for the treatment of wounds and chronic lesions, and for the repairs of damaged joints. Therapeutic gels, creams, and injections with NPs are used to deliver high local concentrations of active agents while minimizing their radius of action. In addition, NPs that have the capability of controlling inflammation, promoting fibroblasts, and scavenging oxidants also dovetail with the standard physiotherapeutic approaches. The adoption of this technology in physiotherapy is a major leap forward in the management of patients in that it leads to improved recovery rates, shorter rehabilitation periods, and treatments specific to individual patients.
